# Understanding consistencies and gaps between desired forest futures: An analysis of visions from stakeholder groups in Sweden

**DOI:** 10.1007/s13280-015-0746-5

**Published:** 2016-01-07

**Authors:** Camilla Sandström, Annika Carlsson-Kanyama, Karin Beland Lindahl, Karin Mossberg Sonnek, Annika Mossing, Annika Nordin, Eva-Maria Nordström, Riitta Räty

**Affiliations:** Department of Political Science, Umeå University, 901 87 Umeå, Sweden; Swedish Defence Research Agency, 164 90 Stockholm, Sweden; Unit of Political Science, Luleå University of Technology, 971 87 Luleå, Sweden; Swedish Defence Research Agency, 164 90 Stockholm, Sweden; SLU, 901 83 Umeå, Sweden; Department of Forest Resource Management, SLU, 901 83 Umeå, Sweden

**Keywords:** Backcasting, Conflicts, Forest policy, Frames, Governance

## Abstract

Conflicting perspectives on forests has for a long time challenged forest policy development in Sweden. Disagreements about forest futures create intractable deadlocks when stakeholders talk past each other. The purpose of this study is to move beyond this situation through the application of participatory backcasting. By comparing visions of the future forest among stakeholder groups, we highlight contemporary trajectories and identify changes that were conceived as desirable. We worked with four groups: the Biomass and Bioenergy group, the Conservation group, the Sami Livelihood group and the Recreation and Rural Development group; in total representatives from 40 organizations participated in workshops articulating the groups’ visions. Our results show well-known tensions such as intrinsic versus instrumental values but also new ones concerning forests’ social values. Identified synergies include prioritization of rural development, new valued-added forest products and diversified forest management. The results may feed directly into forest policy processes facilitating the process and break current deadlocks.

**Electronic supplementary material:**

The online version of this article (doi:10.1007/s13280-015-0746-5) contains supplementary material, which is available to authorized users.

## Introduction

The existence of a variety of partially conflicting perspectives in discussions on forests has for a long time influenced the forest sector and forest policy development in Sweden. The Swedish forest sector, here broadly defined as “the economic, social and cultural contribution to life and human welfare derived from forest or forest based activities” (Gane 2007), has undergone a number of changes over time. The modern forest sector is often described as having evolved in stages, starting with the early forest management stage (1820–1890) and progressing to the sustained yield and silvicultural (1890–1945) and intensive industrial management (1945–1990) stages. The current stage (1990s–2010s) has been given different names, all indicating a shift toward an ecomodern approach to forest management, i.e., a vision implying a synergy between environmental protection and economic growth based on established principles of growth, profit and consumerism, and more lately, to social aspects such as aesthetic and recreational values (Sandström and Sténs 2015).

This evolvement is reflected in the Swedish forest policy which rests upon two equal goals: (i) production of timber (and other products) to ensure a sustainable yield; and (ii) safeguarding the environment. It can also be detected in the Swedish Forestry Act (SFS 1979:429) which regulates management and harvesting on all forest land and rests upon the principle of “freedom under responsibility”. The Act, which include few details and strict regulations gives the forest owners i.e., the many non-industrial private forest owners (NIFP) owning 50 % of the productive forest, and the private forest companies owning 25 %, freedom to choose, within certain limits, how to manage the forest. At the same time, the right of access gives the public statutory rights to freely enter any forest and pursue recreational activities such as to pick berries and mushrooms. A traditional land use that also intersects with forest ownership is the usufructuary right of the aboriginal Sami people, i.e., a civil law term implying the right to let their reindeer graze in the forest within the reindeer management zone, which constitutes approximately 50 % of the Swedish land area (Swedish Forest Agency 2014).

Hence the Swedish forest policy can be described as multi-objective, involving a wide range of stakeholders and their frames, here understood as specific applications of general cognitive commitments, or “thought styles”, acted out in a social context (Perri 6 2005). The variety of frames and how they are expressed in terms of visions and values, however, constitutes a challenge for policy development. Disagreements about forest futures not only create conflicts but also lead to deadlocks when the stakeholders talk past each other, severely hampering policy development (Sandström and Sténs 2015). Although many studies have tried to identify similarities and differences between different stakeholders’ perceptions and visions in order to move the discussion forward, the focus has often been constricted to current policies with an emphasis on immediate implementation rather than policy development (e.g., Beland Lindahl 2008; Kindstrand et al. 2008; Eriksson et al. 2014).

The purpose of this exploratory study focusing on Sweden is to move beyond the current situation by applying a long-term and integrative tool, participatory backcasting, to identify stakeholders’ various desirable forest futures and then to compare these visions in order to highlight contemporary trajectories and identify changes that were conceived as desirable. We suggest that visions could be used to raise general awareness of tensions between different frames, but also to identify consistencies that could provide a basis for compromise. Moreover, our examination of differences and similarities between different desired futures provides an indication of key potential conflicts and synergies that may emerge in the public debate on forests in the years to come.

## Materials and methods

### The backcasting approach

Backcasting involves envisioning, and it has been suggested that creating visions, as well as applying other future oriented tools, can be helpful when building sustainable societies (e.g., Sedlacko and Gjoski 2010; Bazan et al. 2014; Wick and Iwanic 2014). In this study, we used *participatory backcasting* which is a process where stakeholders take an active part (Carlsson-Kanyama et al. 2008; Quist et al. 2011). Backcasting is normally used for future studies when existing trends indicate that development is going in an unfavorable direction (see Dreborg 1996 for the theoretical foundations of backcasting). Backcasting aims at depicting normative scenarios (visions) of desired futures as opposed to futures that are likely to happen, and to work backwards from these futures to the present time in order to determine what measures would be required to reach the desired future (e.g., Vergragt and Quist 2011). Backcasting with its envisioning part has evolved over the years but many studies still lack sufficient details to be replicated (Wick and Iwanic 2014). A number of quality criteria for the visions have been proposed and it has been suggested that backcasting could evolve further by comparing approaches across countries (Vergragt and Quist 2011), and by including social structure and agency to a larger degree than now (Wangel 2011). Regarding the impacts of participative backcasting it has been shown that it may lead to follow-up and spinoff but that it depends on e.g., the degree of stakeholder involvement (Quist et al. 2011). Different approaches exist within participative backcasting, including different methods applied. Quist and Vergragt (2006) have proposed a five-stage model of a generalized methodological framework for participatory backcasting, and in this study, we focused on the first two stages: problem orientation and construction of visions. The work was based on substantial experience of using backcasting for formulating visions about sustainable cities and municipalities (e.g., Carlsson-Kanyama et al. 2008, 2013).

### Selection of stakeholder groups

The selection of stakeholder groups is based on an analysis undertaken to identify who have a stake in the future of Swedish forests. The term stakeholder refers to “all those who affect, and/or are affected by the policies, decisions and actions of the system” (Grimble et al. 1995). Stakeholders can be individuals, social groups or organizations at various levels in society. Our analysis recognizes key stakeholders at multiple levels in the Swedish forest sector. However, the social practices and meanings attached to the forest may vary considerably even among individuals. To allow for an analysis of desired futures, we assume that these meanings can be grouped in accordance with how different stakeholders interpret the world through frames based on experience, knowledge and values (Perri 6 2005; Beland Lindahl 2008). We also assume that, based on these frames, the stakeholder groups desire a specific agenda and certain actions. The selection of stakeholder groups is consequently based on the assumption that there are different frames that influence the visions of the desired forest futures. This approach is similar to the methodology for selecting stakeholders proposed by Cuppen et al. (2010) whereby perspectives are used as a basis for selection.

Based on previous research on stakeholders related to the forest sector (Beland Lindahl 2008, 2015; Beland Lindahl and Westholm 2011, 2012; Beland Lindahl et al. 2013), we identified four groups of key stakeholders with relatively similar understandings of forests and forest management. The first group, Biomass and Bioenergy (BB), includes representatives from organizations with a stake in the extraction of resources (mainly woody biomass) from the forest. This group includes forest enterprises, forest owners, bioenergy enterprises as well as governmental agencies in charge of forest-related issues. The second group, Conservation (C), includes representatives from organizations with a stake in biodiversity protection. This group includes non-governmental organizations and the government’s environmental authorities at national and regional levels. The third group, Sami Livelihood (SL), consists of indigenous Sami organizations sharing the cultural tradition of herding reindeers in the forest. The fourth group of organizations, Recreation and Rural Development (RRD), is a relatively diverse collection of interest groups. We assume that they share frames promoting experiences related to social forest values such as different forms of outdoor recreation and well-being, but also rural development. This group is potentially the least cohesive because of internal tensions between urban and rural values.

The number of stakeholder groups representing each category varied considerably due to both structural and institutional factors related to Swedish forest policy. The degree of institutionalization of the individual stakeholder groups and capacity in terms of resources (time) also affected the incentive or the capacity to participate. In total 52 actors were invited to participate in the project; of these there were 12 organizations which declined to participate primarily due to lack of time, while a couple of them did not consider the project to be of relevance to their organization. A full list of the stakeholder groups can be found in Table 10.1007/s13280-015-0746-5.

### Method for data gathering and analysis

Six workshops were held at the Swedish Defense Research Agency (FOI), Kista in April to June 2014 and two workshops were held at a hotel in Lycksele in 2014 during the backcasting process, two for each stakeholder group. The aim of these sessions was to create the groups’ visions and to sketch the events, including policy measures, necessary to reach those visions. Here, only the visions are discussed as the measures to reach the visions will be further developed within the stakeholder groups. By arranging the backcasting process in the premises of FOI and at a hotel, we ensured a non-biased process, primarily because of the expertise in this type of processes provided by FOI, but also due to the absence of connections to forest policy processes among the FOI researchers.

**Fig. 1:**
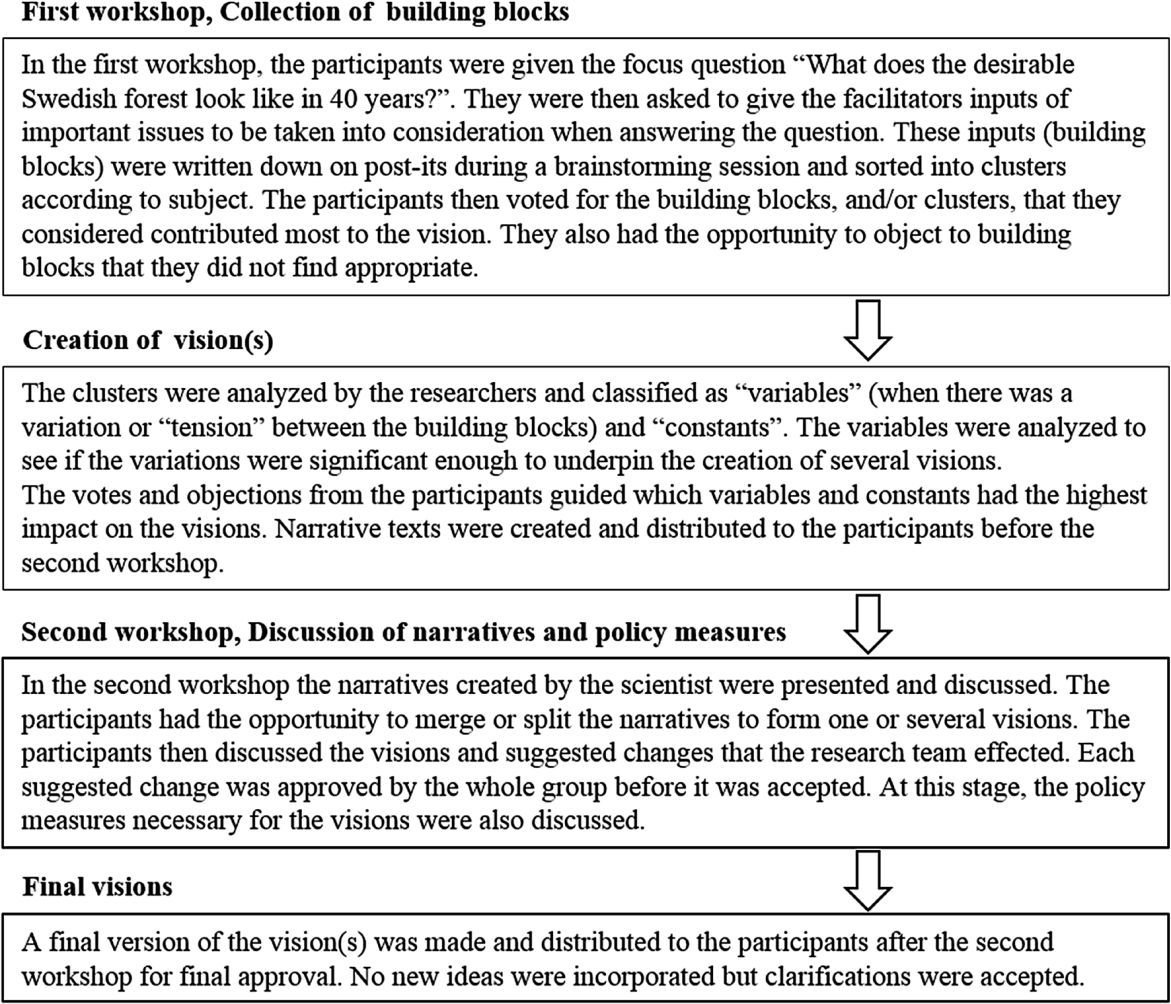
The process for creating the stakeholder groups’ visions, held in the same way for all stakeholder groups

In order to create the visions, the research team prepared and facilitated the workshops, and created drafts and final versions of the visions as they emerged during the discussions (Fig. 1). To make the visions as representative as possible, we aimed for broad participation from the members of the groups and gave the groups ample time to develop and refine their visions. Finally, the visions were analyzed by the research team focusing on central themes of forest policy and practice. In order to compare the visions, all identified themes from each of the visions were listed in a database. The visions were then scanned for quotes that fitted each theme, and these quotes were also entered in the database. Each theme was then analyzed using the quotes; some iteration was included to find the most important and interesting differences and similarities between the visions. The aim was not to be comprehensive but to identify how the different stakeholder groups, based on more or less shared frames, imagined a desirable future.

## Results

We found that three of the four groups of stakeholders were capable of agreeing within their respective group on one common vision of the desired future for the forest. The fourth group, consisting of organizations with a stake in recreation and rural development, could not agree on one common vision of the desired future, and came up with two different visions (Table 1).

**Table 1:** Summaries of the five visions of the desired future of forests from four groups of stakeholders

Stakeholder group which produced it	Summary of content
Biomass and Bioenergy (BB)	Swedish authorities continue the forest policy of “freedom under responsibility” and simplify rules for landowners and forest companies. The forest and its products are a pillar of the Swedish economy, and the production of timber and the added value in the forest sector has increased substantially compared with today. Forest management is efficient, for example, thanks to improvements in technology, and the forests contribute to raw materials to chemical and textile industries as well as to renewable energy
Conservation (C)	Sustainable forestry is carried out on half of the forested land area, while the other half is conserved or used for tourism, hunting and recreation. A lot of people have moved from cities to rural areas and many of them make a living from nature tourism with many customers from abroad. Others work for local manufacturers processing forest products. In the forestry sector, both planting and harvesting is carried out in an environmentally friendly manner
Sami Livelihood (SL)	Society recognizes the rights of the Sami people and gives reindeer herding communities’ crucial power over decisions about land use and land sale in the reindeer management zone. Among other things this means that trees are left to grow old, there is no clear-felling and infrastructure such as roads and windmill-parks are scarce or adapted to the needs of the reindeer. As a result, the Sami people can sustain themselves on reindeer management all-year round
Recreation and Rural Development (RRDa)	There is a new Swedish forest policy that ensures that forestry considers ecological and social values, and gives local stakeholders an influence over large forestry companies. Continuous cover forestry is used in most forests and only domestic tree species are planted. There are many local manufacturers processing forest products and many companies that promote Swedish forest tourism. Biomass from the forest is an important part of society’s energy transition from fossil fuels
Recreation and Rural Development (RRDb)	The Swedish authorities recognize that the forest owners are best suited to care for the forests by themselves, and therefore avoid imposing regulations. The owners manage their forests carefully, resulting in species-rich forests with high recreational value; the forest owners can make a good living by charging entrance fees to interesting areas. A substantial part of the forest is owned by small local companies which produce according to local demand and often process products. Education and research about forestry is prioritized

Our results show that all four stakeholder groups identified several common themes that they considered important for the future use of forests. These themes related to fundamental values, property rights, forest governance, forest management and conservation, and processing and industrial use of raw materials.

### Values related to forests

Previous research has shown that certain values and beliefs tend to go together, while others tend to be opposed to each other. This is particularly visible in the contested area of natural resource governance, where some stakeholders are concerned with the direct or extractive use of natural resources for personal or commercial purposes and others are interested in the indirect use and conservation for its own sake (O’Neill 1992; Owen et al. 2009). This division between instrumental and intrinsic values also appears in this study. The view that the forest has intrinsic values was most pronounced in the Conservation (C) group. This view emerged from the desire to “*respect of nature’s limit*” and the belief that *“ecological values are the basis for society and human welfare and therefore for economic and social values”* (C group). The Recreation and Rural Development group which was split into two groups (RRDa and RRDb) partly based on the distinction between intrinsic and instrumental values, also highlight the fact that *“the forest is increasingly seen as something with intrinsic values, not as something that is only of use as property or for consumption”* (RRDa group).

The instrumental values were mainly represented by the Biomass and Bioenergy (BB) group as well as one part of the Recreation and Rural Development (RRDb) group, although each had very different motives. The BB group focused in particular on the economic importance of the forest, forestry and related industries, and how the sector contributes to general welfare in Sweden, while the instrumental view reflected in the RRDb emphasized in particular multiple values and the responsibility of the individual forest owner: *“*[*B*]*y developing the services and products that the forest can contribute with … most forest owners today practice sustainable economic management”*.

The Sami Livelihood (SL) group in turn departs from intrinsic values when they state that *“clean soil, clean air and clean water, together with a powerful Sami position and a strong respect for nature, have contributed to the conditions for the continuance of Sami traditional livelihoods”*. The intrinsic view on forests could thus actually contribute to more instrumental values—in this case the needs of the reindeer herding industry.

Another example of how intrinsic and instrumental values influenced the visions is in the area of sustainable forestry. The C group viewed ecological values as the basis for economic yield, and thereby saw a need for sustainable methods, the BB group saw it the opposite way around and stated that economic yield was the basis for sustainable forestry. The BB group also viewed the forest as an instrument to “*… mitigate climate change by utilizing the forest to bind carbon dioxide and by replacing fossil fuel and climate*-*impacting material with biomass”*. This view is to some extent shared by both RRD groups that state that “*[t]he forest contributes to a sustainable energy conversion, for example by locally adapted withdrawal of biofuel”*.

### Property rights

The Swedish forest land is covered by several layers of property rights (public and private ownership, hunting rights, rights to public access, and the right of the Sami to practice their traditional way of life) and the different groups emphasized or downplayed property rights according to whether they would strengthen their own visions. Paradoxically though, the different visions actually led to similar outcomes in terms of conditions for forest entrepreneurship. The BB group emphasized strong private property rights and envisioned new entrepreneurial opportunities in the future. The potential for diversity and employment based on products and services from forests was also addressed by the RRDb group: “*Today the forest is often owned by small and locally based companies who choose tree species according to interest and needs and whose companies generate revenues that stay in the countryside and give employment there”*. The SL group emphasized the role of a vibrant cultural landscape where traditional Sami activities are common, widely accepted and respected throughout Sweden. The C group anticipated a similar future with a multiplicity of small forest companies instead of the current situation where a few large ones dominate.

This paradoxical unity of future entrepreneurship in the forest does not extend to views on the right to public access, which in the desired visions can either be seen as an obstacle to development for entrepreneurship *or* a prerequisite for the same. The RRDb group advocated a far-reaching weakening of the right to public access in forests in order for landowners to generate more income from their land. The other groups either wanted to maintain the right to public access as it is today –*“In Swedish society the right of public access stands strong”* (C group), and *“The right of public access is applied and is continuously exerted”* (RRDa group)—or make only minor moderations to impede misuse by commercial actors –*“The right of public access applies but aberrations in the form of littering and overuse have disappeared”* (BB group).

### Governance and collaboration

One of the most marked differences between the visions concerned how and by what means the stakeholder groups wanted the future forests to be governed. While some promoted centralization and stronger regulations, others supported much greater decentralization and privatization compared with the current governance model.

While the BB group largely wanted to maintain the status quo for the foreseeable future with the policy based on *“freedom under responsibility”*, the RRDb called for more leeway for the individual landowner. The SL group went a step further by envisioning the Sami having *“veto rights concerning intrusions on all land within the reindeer husbandry area”*, and decentralized governance in the all-year round area close to the mountain region, where the Sami rights to land are particularly strong today. The other groups anticipated a completely different view of the future governance of forests; they saw the government having a strong role in parallel with a diversity of actors in the governance process. The RRDa vision included a strong government with *“overall responsibility for forest policy—decisions are based on dialogue and collaboration between authorities, landowners and other users”.* The C group suggested a future which goes beyond the current situation of mere participation for civil society actors, and thus imagined a power shift in favor of the public. With regard to collaboration over forest policies, all but one group (the RRDb) envisioned a much more relaxed climate for debate than today. Expressions that convey this ranged from *“discussions in a respectful way”* (C group), to *“the dialogue is constructive”* (RRDa group) and *“there are no longer any conflicts between forestry and reindeer herding”* (SL group).

### Forest management and conservation

All groups, except the BB one, suggested substantial changes in forestry practices in their visions primarily to meet current and future environmental concerns. These changes excluded many of the measures associated with contemporary Swedish forestry such as monocultures, soil scarification, stump extraction, clear-felling, and the use of non-native tree species. Instead, these groups promoted policies favoring deciduous tree species, new methods for logging and the planting and use of native tree species. Furthermore, in the visions developed by the C group, forestry with the expressed purpose of extracting timber and products such as pulpwood, bioenergy and chemical substances was only conducted on 50 % of the forest land. Nature and wildlife management, recreation, tourism and outdoor activities, and reindeer husbandry were, however, conducted on all land, and it was of *“great importance that all types of land use should be sustainable from several aspects, such as maintaining biodiversity, social values and cultural values over time and space and to maintain a long*-*term productive capacity”* (C group).

In contrast to the other groups, the future vision of the BB group focused on formulated goals for biomass production. Their vision was that 150 million m^3^ of timber per year would be harvested—approximately a 75 % increase compared to today. This would be achieved by better land consolidation, technological development and more efficient forest management. In terms of conservation the BB group relied on market-driven instruments such as forest certification designed to enable diverse forest management for all types of forest owners and ensure that *“[L]and use is differentiated, which means for example that parts of the forest can be operated intensively while other parts are managed with special considerations”.* The proffered means to achieve these changes differed between the groups: suggestions included the use of governmental regulation (C group) and increased responsibility and room for action by private landowners (RRDb group). With regard to the latter it was felt that due to the many different managerial approaches among private landowners increased freedom of action would lead to *“a high diversity with deciduous and mixed forests, a variety of ages among the trees and many different species”* (RRDb group).

The SL group envisioned that the rights of the Sami people were respected and that forestry within the reindeer husbandry area was adapted to the needs of reindeers. Adaptation to reindeer husbandry would include a range of measures such as longer rotation periods and thinning to promote lichen growth facilitating reindeer migration. The SL group also emphasized that forestry should not manipulate nature to grow in *“unnatural”* ways: *“Today we are not deceiving the forest in a way that is not in harmony with nature”*.

### Processing and industry

All groups, except the SL one (which primarily focused on the reindeer herding industry), included many new opportunities for forestry-related processing and industry in their visions. However, the profile of these new opportunities differed: the C group and the two RRD groups were very definite about opportunities related to nature tourism which had expanded significantly in their visions. The C group also highlighted the potential to develop new working opportunities and even new lifestyles in rural areas since *“[A]n increasing number of people live outside the bigger cities and earn a living from natural resource management in Sweden today”.* The group viewed the nature tourism sector as potentially offering a lot of new jobs.

In contrast, the BB group did not mention tourism opportunities in their vision but rather focused on tangible products such as chemicals, fibres, and renewable energy production. These products were also emphasized by the RRDb group. All groups, however, included an increased market for wood in their visions, envisaging a society with increasing demands for renewable materials—demand for wood used in house construction, for example, would be heavy.

## Discussion

In this study, we used visioning, as part of a participative backcasting process, to explore differences and similarities between stakeholders’ desired forest futures. We grouped stakeholders that shared similar frames, which allowed us to, more thoroughly explore their specific agendas for the future than if we would have grouped stakeholders with different frames. Our analysis of the groups’ desirable futures may feed into policy making, contributing to a deeper understanding of the forest sector among decision-makers. Our focus on the similarities and differences clarifies both areas in which there is consensus among stakeholders for a given development, and areas in which current or new conflicts between stakeholders may hamper any development.

Our study confirmed the well-known divide between instrumental and intrinsic values. The instrumental values were mainly expressed by forest owners and forest industry in the BB group, but also by rural development interests (part of the RRD group) emphasizing the multiple use of forest resources from a utilitarian perspective. The intrinsic values accentuated by the other groups highlighted other functions that were seen as important, e.g., biodiversity and other services provided by forests. Given how this division, which has long been the most dominant feature in the Swedish forest policy debate, clearly permeates the desired forest futures, we can expect it to persist for some time to come.

However, our study also shows that this gap is to some extent challenged by slightly different values, usually referred to as the forest’s social values, offering both potential synergies between the groups and increased tensions. While research on forests’ social values has primarily focused on such in relation to urban environments (Lindhagen and Hörnsten 2000; Olsson 2014), the social role of forests in rural contexts has largely been neglected. Our results point in a direction of desired change from this exclusive urban focus to forests’ social values as a possible future source of employment in rural areas, emphasized in all the desired futures. This includes rural-based small-scale entrepreneurship ranging from the development of new wood products, reindeer husbandry, tourism and recreation by offering the growing urban population a retreat in rural areas. Although social values are highlighted in all of the desirable futures—potentially making it easier to establish synergies between the different futures—it is also possible to find significant differences of how social values are expressed in the visions There is a distinct division between development based on traditional extractive forestry and wood-based product development and development based on forest aesthetics, tourism and recreational values. Once again we may expect a tension primarily between traditional forest management for biomass yield and the possibility of developing businesses based on ecosystem services other than biomass production. This tension may be related to a wider shift in value orientations stemming from a combination of the following: demographic change related to urbanization; the emergence of new recreational and aesthetic preferences among the public; and the profound economic changes that may make society less directly dependent on its natural resource base (Owen et al. 2009).

Another important division between groups that emerge in the different desired futures is how the forest as a resource is to be governed. There is considerable tension between competing views on this important issue: some—invoking the principle of freedom under responsibility—view forests as private property; others view the forests as a common resource and assert parallel property rights such as usufructuary rights and the right of public access. This tension is also reflected in the various approaches to the governance of future forests; the BB group largely want to keep the situation as it is, while the other groups would like to see changes both in terms of greater centralization of power to the state (C and RRDa groups) and, conversely, the decentralization of power to forest owners (RRDb group) or indigenous forest users (SL group).

Although the different visions are characterized by a number of divisions and tensions, there is also great potential for synergies in particular regarding how forests can contribute to innovation and job creation through new value added forest products. All the visions envisage a society with increasing demands for forest resources both for industrial but also for recreational use. This requirement is linked to desires for more variation in the forested landscape and thus also an identified need to a wider variety of silvicultural practices, including possibilities for zoning or differentiation of management at different scales. These desires could provide a fresh start for the discussions in the newly instigated National Forestry Programme.

## Concluding remarks

We used participative backcasting to formulate visions of the future Swedish forests for four different stakeholder groups. The selection and grouping of stakeholders were based on previous studies resting on frame analysis. Our approach was novel and proved to be successful in identifying different groups of stakeholder groups promoting similar forest values and desired futures (based on either more instrumental or intrinsic values), but also groups with completely different visions for the future, such as a more post-productive visions. As a result of the high degree of participation, the participatory process appeared to be highly appreciated by the involved stakeholders and may be further used to promote capacity building within each of the stakeholder groups in terms of a commonly expressed vision that can be used also in relation to the other groups. The results both in terms of the visions, but also our analysis may feed directly into current forest policy processes and help policy makers to prioritize between issues, not the least since we found areas where compromises may be possible. The study also support long-term thinking by expanding the time horizon of policy making, which gives access to more options for the future and thus flexibility of governance. Hence, we believe our study may facilitate current policy processes and potentially break current deadlocks in the debate on the future of forests.

## Electronic supplementary material

Supplementary material 1 (PDF 404 kb)
