# Supplementary material for: Understanding consistencies and gaps between desired forest futures: An analysis of visions from stakeholder groups in Sweden
Source: Ambio. 2016 Jan 7;45(Suppl 2):100–8. doi: 10.1007/s13280-015-0746-5 (PMC4705062; doi:10.1007/s13280-015-0746-5)
Supplement: Supplementary file 1 — Supplementary material 1 (PDF 404 kb) [file 13280_2015_746_MOESM1_ESM.pdf]

Ambio

Electronic supplementary material

This supplementary material has not been peer reviewed

Title: **Understanding consistencies and gaps between desired forest futures: An analysis of visions from stakeholder groups in Sweden**

Authors: Camilla Sandström, Annika Carlsson-Kanyama, Karin Beland Lindahl, Karin Mossberg Sonnek, Annika Mossing, Annika Nordin, Eva-Maria Nordström, Riitta Räty

Table S1 List of stakeholder groups (based on Beland-Lindahl 2015)

Frames			
Biomass and bioenergy frame	Conservation frame	Sami livelihood frame	Rural development and recreation frame
LRF Skogsägarna	Naturskyddsföreningen	SSR	Hushållningssällskapet
Sveaskog	WWF	Renägar-förbundet	Hela Sverige ska leva
SCA	Jordens Vänner	Same ätnam	Sveriges hembygdsförbund
Bergvik skog	Fältbiologerna	Saminourra	Sportfiskarna
Stora Enso	Framtidsjorden	Sametinget/ Parlamentet	Svenska jägareförbundet
Holmen	Global Rättvisa Nu	Sametinget/ Myndigheten	Jägarnas riksförbund
Preem	Klimataktion		Friluftsfrämjandet
Neova	Länsstyrelsen		Svenska orienteringsförbundet
Svebio	Norrbotten		Tillväxtverket
Skogsindustrierna	Länsstyrelsen Kalmar		
Skogsstyrelsen	Naturvårdsverket		

Energimyndigheten

Spillkråkan

.
